# Using information and communication technology learnings to alleviate social isolation for older people during periods of mandated isolation: A review

**DOI:** 10.1111/ajag.13041

**Published:** 2022-02-09

**Authors:** Emily Todd, Bronwyn Bidstrup, Allyson Mutch

**Affiliations:** ^1^ School of Public Health The University of Queensland Herston Queensland Australia; ^2^ Council on the Ageing (COTA) Queensland Brisbane Queensland Australia

**Keywords:** aged care facility, communication media, physical distancing, social isolation, social participation

## Abstract

**Objective:**

To examine the effectiveness of information and communication technologies (ICTs) in reducing social isolation in older people and draw recommendations from previous literature appropriate for informing ICT use in future mandated periods of isolation.

**Methods:**

A systematically conducted review of key databases to identify studies investigating ICT interventions that targeted social isolation or loneliness among older people.

**Results:**

Fifteen articles were identified. All articles used ICT as an intervention for targeting social isolation with varying results. Most studies reported positive impacts on social isolation, but this was identified more in self‐reporting compared to changes in baseline measures. The types of ICT used included videoconferencing, Internet‐based applications and purpose‐designed applications. A number of factors were also identified throughout the studies that impacted uptake that should be considered when implementing ICT.

**Conclusions:**

Overall, we found evidence of ICT improving social connectedness of older people to some extent although more rigorous research in future is needed. Recommendations from previous literature highlight the importance of including older people in purposeful design, engaging families and support networks, and providing ongoing ICT training and support so that systems and skills are in place for future periods of mandated isolation. The literature also warns us not to rely on ICT as the only avenue for social interaction either during or outside periods of social distancing.

**Policy Impact**

Many technological adaptions that occurred during the COVID‐19 pandemic have remained and this paper highlights a population, already at risk of social isolation, who may need additional support when connecting through technology. These recommendations can be used to support the best practice for connecting older people during and after COVID‐19.

**Practice Impact**

These recommendations aim to empower older people through building and maintaining social connections without the need for face‐to‐face contact. During periods of mandated isolation, when these adaptions are necessary, the use of family involvement, training, ongoing support, and simplistic technology can improve uptake and limit feelings of social isolation.

## 1 INTRODUCTION

Loneliness, or the perceived feeling of social disconnection, along with social isolation, the state of reduced social interaction and limited social networks, is an area of critical concern across our ageing population.^1^, ^2^, ^3^ In 2018, around 8.0% of Australians older than 65 experienced social isolation, linked to limited social connections and support networks; 13.1% of 65‐ to 69‐year‐olds, and one in five 75‐year‐olds specifically identified deeper emotional episodes of loneliness.^1^, ^4^ Social isolation is commonly associated with poor mental and physical well‐being.^5^ For older people, social isolation is frequently connected to dwindling networks, living alone or in a residential aged care facility (RACF).^3^, ^6^ Irrespective of age, Holt‐Lunstad et al.^7^ found experiences of loneliness and social isolation increased the chances of premature mortality by almost a third.

In Australia, the majority of those older than 65 live in the community, while only 5.2% live in a RACF.^8^ Of those living at home, 27% live alone, but this figure increases with age: around one in three older than 75 live alone.^8^, ^9^ Efforts to prevent or minimise social isolation among older adults have seen researchers investigate initiatives targeting those residing independently and people living in RACFs.^10^ Interventions have included group activities, companion pets, robots, technology and mentoring.^10^, ^11^, ^12^ A systematic review evaluating different interventions found that irrespective of the activity, interventions conducted in groups with participants completing an activity together were the most effective at reducing social isolation.^10^ Despite the benefits of interactive interventions targeting isolation, it may not always be possible to engage people in groups. Recent social restrictions stemming from the COVID‐19 pandemic bear witness to the impact of mandated periods of isolation that render interactive programs untenable and dictate the need to identify alternative methods to address isolation.^13^

COVID‐19 emerged in Australia in early 2020, pausing gatherings, commuting, travel and socialising.^13^, ^14^ In 2020, Australia minimised the early spread of this virus, but to date, 2117 people have died (16 December 2021): 80% were older than 70.^14^ The virus has disproportionately affected older people, with the integration of physical distancing into everyday lives placing more strain on those experiencing social isolation.^15^, ^16^ During the pandemic, many RACFs have enforced strict lockdown measures, banning outside visitors and mandating physical distancing between residents.^15^ People older than 70 living in the community were strongly advised by public health officials to stay at home wherever possible and to have groceries and medications delivered.^17^ The implementation of these restrictions led many to turn to technology to maintain contact with friends, family and others.^18^

Researchers have investigated the potential for technology to be used to connect older people, with a number of systematic reviews examining information and communication technology's (ICT) effect on social isolation in older people.^3^, ^19^, ^20^, ^21^ Noone et al. investigated the effectiveness of videoconferencing, concluding that the absence of a significant body of current research (three papers reviewed) undermined their ability to draw conclusions about its ability to reduce social isolation.^19^ Three additional systematic reviews, conducted by Chen and Schulz,^3^ Baker et al.^21^ and Ibarra et al.,^20^ investigated the use of ‘off‐the‐shelf’ technologies (e.g., email, Internet, apps, telephone and videoconferencing) and some purpose‐designed technologies. Chen and Schulz's extensive review included training interventions, telehealth, and interventions connecting people to virtual pets, along with apps, social media, telephones, games and email.^3^ They found most ICT interventions (video calls, Internet and telephoning) had a positive impact on social isolation, but gains were short‐term and frequently not measurable after 6 months. Baker et al.,^21^ who also included ICT training interventions in their review, found touchscreens and Internet‐based applications (e.g., social networking sites) were most frequently investigated, but the diversity of methodologies prevented comparisons of the effectiveness of ICT applications: this was a finding reiterated by the other reviews.^3^, ^20^ Ibarra et al.^20^ also found all studies reported positive impacts of ICT on social isolation, but most included face‐to‐face activities designed to improve digital skills and emphasised the essential role technical support and training played in uptake.

In the light of the challenges associated with comparing the vast diversity of ICT applications, Chen and Schulz argue a more effective approach to simply reviewing the literature is to draw out broader recommendations that guide the identification and implementation of effective forms of ICT that meet the needs of older people.^3^ Certain circumstances can influence ICT’s implementation for older people, particularly during mandated periods of isolation, such as lockdowns during the COVID‐19 pandemic, or during other periods of isolation (e.g., hospitalisation). Given the restrictions imposed by mandated isolation, it is essential to identify interventions not reliant on face‐to‐face training. The following review aimed to draw recommendations regarding the effective integration and use of ICT within the context of mandated isolation. Through this lens, we systematically reviewed the literature to examine whether ICT can be used to reduce social isolation among older people during periods of isolation. In addressing this question, we also aimed to identify recommendations relevant to technology use for older people during times of social distancing. Such recommendations will potentially assist RACFs and other institutional settings to support residents.

## 2 METHODS

### 2.1 Search strategy

A literature review was systematically conducted using the Preferred Reporting Items for Systematic Reviews and Meta‐Analyses (PRISMA) protocol guidelines.^22^ In September 2020, 6 databases were identified in collaboration with a senior librarian and searched, including PubMed, Embase, CINAHL, Cochrane (Cochrane Reviews), RURAL and PsycINFO. A search strategy was developed using key terms: ‘older people’, ‘ICT’, ‘social connection’ and ‘social isolation OR loneliness’ (see Table 1). The use of synonyms and MeSH terms expanded the search's reach; for example, ‘elder*’, ‘ageing’, ‘aging’, ‘aged’, ‘senior’, ‘retir*’, ‘old person’, ‘old people’ and ‘geriatric’ were also searched alongside ‘older people’. The search strategy included the desired outcome (social connection) and the target (social isolation or loneliness) to increase the likelihood of finding interventions. Additionally, the reference lists of four systematic reviews were reviewed to identify relevant research.^3^, ^19^, ^20^, ^21^

**TABLE 1:** Search strategy example

Older people	Social isolation	Information and communication technology	Social connection
"elder*"[TIAB] "Ageing"[TIAB] "Aged"[Mesh] "Senior*"[TIAB] "Retir*"[TIAB] "Old person" [TIAB] "Old people" [TIAB] "Older person"[TIAB] "Older people"[TIAB] "Geriatric*"[TIAB]	"Isolat*"[TIAB] "Remot*"[TIAB] "Social isolat*"[TIAB] "Quaratin*"[TIAB] "Reclusiv*"[TIAB] "Social exclu*"[TIAB] "Lone*"[TIAB] "Alone"[TIAB] "Seclu*"[TIAB] "Withdraw*"[TIAB] "Social Isolation"[MeSH]	"Technology"[MeSH] "Digital*"[TIAB] "Internet"[MeSH] "Comput*"[TIAB] "Device*"[TIAB] "videoconferenc*"[TIAB] "Video conferenc*"[TIAB] "Zoom"[TIAB] "Skype"[TIAB] "Microsoft Team*"[TIAB] "Facetim*"[TIAB] "Virtual"[TIAB] "ICT"[TIAB] "Information Communication Technolog*"[TIAB] "Information technology*"[TIAB] "Tablet*"[TIAB] "Messag*"[TIAB] "Instant Messag*"[TIAB] "Smart phon*"[TIAB] "telephon*"[TIAB] "iPhon*"[TIAB] "laptop*"[TIAB] "iPad*"[TIAB] "Mobile Applications"[MeSH] "User‐Computer Interface"[MeSH] "Social Networking*"[MeSH]	"Social participation*"[MeSH] "Friends"[MeSH] "Social connect*"[TIAB] "Social engag*"[TIAB] "Social inclusion"[TIAB] "Companion*"[TIAB] "Family Relations"[MeSH]

### 2.2 Inclusion and exclusion criteria

Inclusion criteria included peer‐reviewed research investigating the use of an ICT intervention to target social isolation or loneliness among people 65 years and older. All methods were included, and studies published in English and from an OECD country were selected. Articles were excluded if they were published before 2000 to ensure ICT analysed was current.^3^ Additionally, our research aimed to investigate ICT as a means of connecting people who were physically distanced, in line with notions of mandated isolation; therefore, the papers that used in‐person face‐to‐face training or mentoring were excluded.

**FIGURE 1:**
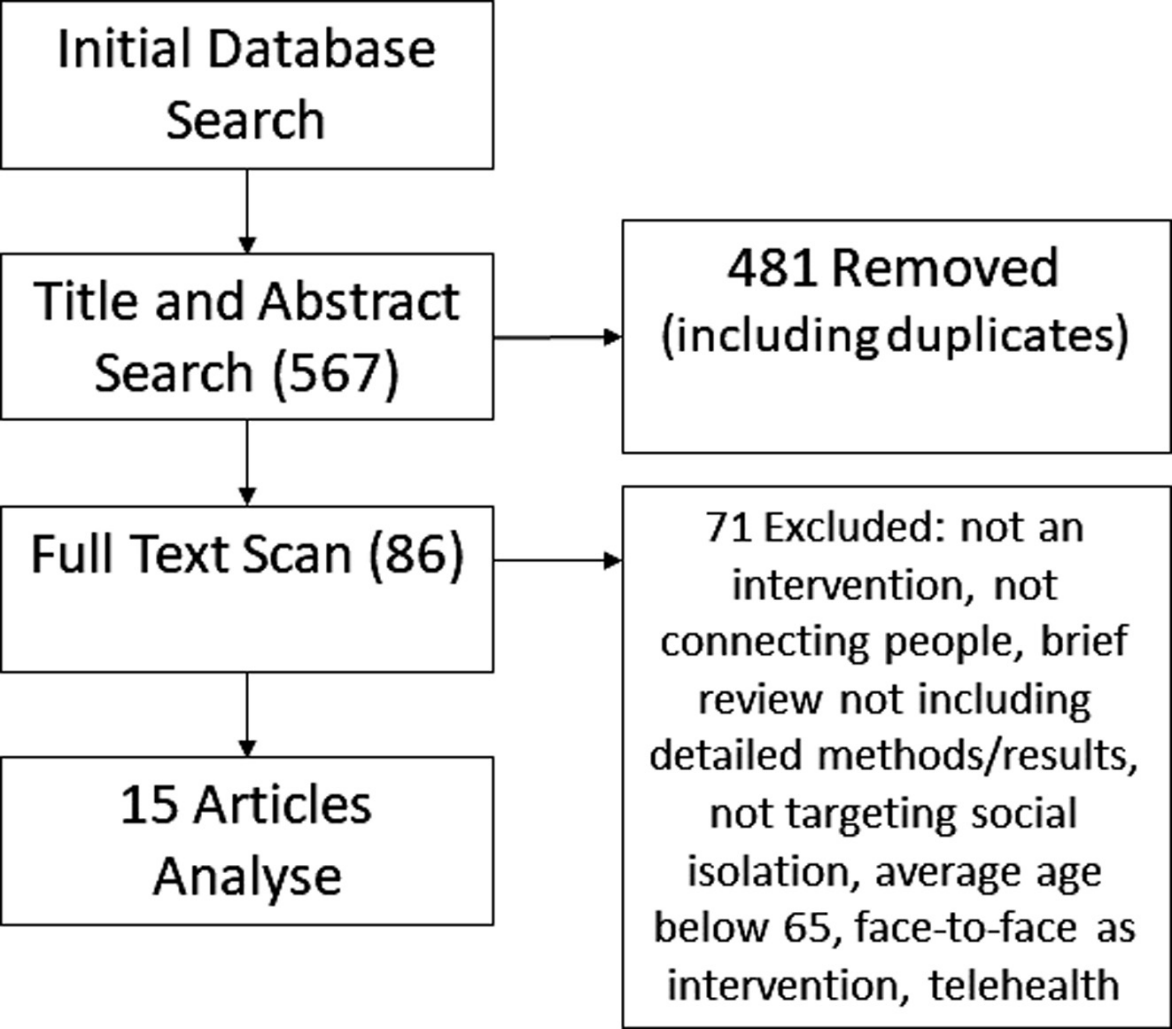
Literature review article inclusion process

After searching the databases and scanning reference lists, ET screened titles and abstracts of identified studies. Following the removal of duplicates and articles not meeting the inclusion criteria, 19 articles were identified for full review. AM reviewed these articles, and a further four papers were excluded following discussion. Reasons for exclusion included papers that did not: involve an intervention, connect people and provide detailed methods or results, along with papers that included participants younger than 65 years or involved face‐to‐face interventions. Fifteen papers were included for final review (see Figure 1).

### 2.3 Quality assessment

Included papers were reviewed for quality using the Mixed Methods Appraisal Tool (MMAT).^23^ The MMAT was selected based on its ability to support quality appraisal of a range of methods including qualitative, quantitative and mixed‐methods studies. Two reviewers reviewed the studies using the MMAT, assessing against criteria such as the measure's appropriateness for answering the research question and the interpretation of results once data were integrated, to provide an overall score based on the star rating set by Hong et al (5 star was the highest quality rating).

### 2.4 Analysis

Due to the high degree of heterogeneity across the studies (variety of settings, participants, ICT interventions and outcome measures), a narrative synthesis was used to analyse the results.^24^ Data extracted for analysis included the effect of the intervention, its size and key findings.^24^ Research questions, method summaries and outcomes were also tabulated and used to identify what the intervention was targeting and its effect (social isolation, loneliness or social connectedness). Participant characteristics, country, setting and ICT intervention were also included. Findings were also noted to inform recommendations. Once tabulated, recurring themes, outcomes or processes (eg incorporation of older people in design) were noted to inform recommendations.

## 3 RESULTS

### 3.1 Study characteristics

Fifteen studies published between 2007 and 2020 were included in the review (see Table 2); eight were published between 2018 and 2020. Studies took place across eight countries: the USA, the UK, Canada, Brazil, Australia, Italy, The Netherlands and Taiwan. Residential aged care facilities (6) were the most common settings for the interventions. Five studies investigated participants living at home: four of the five involved participants living independently.^25^, ^26^, ^27^, ^28^ One study took place in a palliative care unit,^29^ and three did not identify the setting.^30^, ^31^, ^32^

**TABLE 2:** Characteristics of research

Study (year)	Study design	ICT	Setting	Participants	Outcomes
Baez et al. (2017)^27^	Matched randomised pilot study	Internet‐based and applications for phones and tablets	In home	65+, independent‐living, self‐sufficient, non‐frail	Positively impacted loneliness
Barbosa Neves et al. (2019)^38^	Mixed‐methods study	Applications for phones and tablets	RACF	Residents at risk of isolation	Most (83%) found the technology increased social interactions, only 33% felt more socially connected
Cattan, Kime and Bagnall (2010)^32^	Mixed‐methods study	Telephone		Older people	Alleviated feelings of loneliness and social isolation
Czaja et al. (2018)^25^	Mixed‐methods study	Internet‐based application	In home	65+, living independently, English speaking	Sustained improvements for loneliness and social isolation
Fokkema and Knipscheer (2007)^28^	Mixed‐methods study	Internet‐based application	In home	Living independently, reduced opportunities to leave the house, not currently using a computer or the internet	Reduction in loneliness, largest impact on more highly educated people and those who were most lonely at baseline
Guo et al. (2017)^29^	Mixed‐methods study	Internet‐based application	Palliative care unit	Palliative care patients and their family members	Most (89%) of participants felt more connected, closer and calmer
Mellor, Firth and Moore (2008)^39^	Pilot study	Internet	RACF	Older people residing in a RACF	Overall improvement in social connectedness, and initial spike after 3 months that dropped after 6 and 12 months
Mountain et al. (2014)^33^	RCT	Telephone	In home	75+, living at home (independently or with others), English speaking	Negligible difference in overall loneliness
Office et al. (2020)^30^	Qualitative study	Telephone		Older people at risk of social isolation during COVID‐19	Student volunteers were interviewed after one‐off calls. Recalled older adults appreciating having someone to talk to
Seelye et al. (2012)^26^	Pilot study	Videoconferencing	In home	Independently living older people	Most (85%) participants responded positively and saw potential for technology to improve social isolation
Tsai, Cheng, Shieh and Change (2020)^35^	Quasi‐experimental study	Videoconferencing	RACF	60+, no smartphone videoconferencing experience, friends or family members willing to participate	Significant positive effects on loneliness. Frequency of ICT use decreased over time, while the length of calls increased
Tsai and Tsai (2011)^36^	Longitudinal quasi‐experimental study	Videoconferencing	RACF	60+, no smartphone videoconferencing experience, friends or family members willing to participate	Improved perceived loneliness at 3, 6 and 12 months. ICT use decreased slightly over time
Tsai, Tsai, Wang, Chang and Chu (2010)^34^	Quasi‐experimental study	Videoconferencing	RACF	60+, access to wireless Internet	Reduced loneliness after 1 week and 3 months. Intervention group required social connections to communicate with, not required of the control group
Van Dyck et al. (2020)^37^	Qualitative study	Telephoning	RACF	RACF residents, able to hold telephone conversation	Student volunteers interviewed and personally felt more socially connected from experience. RACF residents felt socially isolated prior to the pandemic, only exacerbated through lockdowns. Older people looked forward to recurring phone calls
Zaine et al. (2019)^31^	Case study	Applications for phones and tablets		Two older people were recruited who then invited preexisting social connections to participate. One: father and two children. Two: three female friends	Strengthened social connectedness, inspired deeper conversations and increased frequency of interactions

Abbreviations: ICT, information and communication technologies; RACF, residential aged care facility; RCT, randomised controlled trials.

The studies included a range of methods including two randomised controlled trials (RCTs),^25^, ^33^ controlled quasi‐experimental study^28^, ^34^, ^35^, ^36^ and qualitative research.^30^, ^37^ Six collected mixed‐methods data.^26^, ^28^, ^29^, ^32^, ^38^, ^39^ Only 10 studies used standardised instruments (the UCLA Loneliness Scale, Short Form [36] Health Instrument, DeJong Gierveld Loneliness Scale and Quality of Life Scale^28^, ^29^, ^35^) to measure changes in isolation and loneliness.^25^, ^27^, ^28^, ^29^, ^33^, ^34^, ^35^, ^36^, ^38^, ^39^ The most common scale used to measure social isolation was the UCLA Loneliness Scale.^27^, ^29^, ^33^, ^34^, ^38^ The overall methodological quality of the papers included was relatively poor for the quantitative studies, with some higher methodological quality represented among the qualitative and mixed‐methods studies (see Table 3).

**TABLE 3:** Quality assessment using MMAT

	Qualitative					RCT					Quantitative					Mixed‐methods					Overall quality
Item number	1.1	1.2	1.3	1.4	1.5	2.1	2.2	2.3	2.4	2.5	3.1	3.2	3.3	3.4	3.5	5.1	5.2	5.3	5.4	5.5	
References																					
Baez et al. (2017)^27^						Y	Y	Y	N	Y											****
Barbosa Neves et al. (2019)^38^	Y	Y	Y	Y	Y						Y	Y	Y	N	Y	Y	N	N	N	Y	***
Cattan, Kime and Bagnall (2010)^32^	Y	Y	Y	Y	Y																*****
Czaja et al. (2018)^25^						U	Y	Y	Y	Y											****
Fokkema and Knipscheer (2007)^28^	Y	Y	Y	Y	N						U	Y	N	N	Y	Y	N	N	Y	N	**
Guo et al. (2017)^29^	Y	Y	Y	Y	Y						U	Y	Y	N	Y	Y	Y	Y	Y	U	****
Mellor, Firth and Moore (2008)^39^	Y	Y	Y	Y	U						N	Y	N	N	Y	Y	Y	Y	Y	N	***
Mountain et al. (2014)^33^						Y	U	N	N	N											*
Office et al. (2020)^30^	Y	N	Y	Y	N																***
Seelye et al. (2012)^26^	Y	N	N	Y	U																**
Tsai, Cheng, Shieh and Change (2020)^35^						Y	U	N	N	N											*
Tsai and Tsai (2011)^36^						U	U	N	N	N											U
Tsai, Tsai, Wang, Chang and Chu (2010)^34^						U	U	Y	N	Y											**
Van Dyck et al. (2020)^37^	Y	N	N	N	N																*
Zaine et al. (2019)^31^	Y	Y	Y	Y	Y																*****

Abbreviations: N, criteria not satisfied; RCT, randomised controlled trials; U, satisfaction unclear; Y, criteria satisfied.

*Satisfies 20% of MMAT criteria.

**Satisfies 40% of MMAT criteria.

***Satisfies 60% of MMAT criteria.

****Satisfies 80% of MMAT criteria.

*****Satisfies 100% of MMAT criteria.

### 3.2 Participant characteristics

Sample sizes ranged from 6 to 300. Participants were recruited through convenience sampling within facilities, advertising and participants involved in previous studies. Three included participants identified as at risk of social isolation or loneliness by family or staff.^30^, ^37^, ^38^ Most studies required a minimum age of 65, while others reduced this minimum to 60. The lowest average age was 66 and the highest 82. Throughout the studies, ICT was used to connect older people to friends, family members, spouses, group members and students. Many studies had significantly higher proportions of female participants.^25^, ^31^, ^33^, ^36^, ^38^ Attrition rates were high, with dropout or non‐adoption of technology often due to not perceiving a need for the technology, cognitive decline or death.

A number of studies included findings outlining the experiences of those who had connected with older people through ICT (eg family members, spouses, friends and students).^26^, ^29^, ^31^, ^37^

### 3.3 Overall results

Most studies (12/15) reported positive impacts on social isolation or loneliness based on self‐reporting^26^, ^30^, ^31^, ^37^ rather than changes in baseline measures.^25^, ^33^, ^38^ Of those who did not report positive outcomes, Barbosa Neves et al.^38^ used a purpose‐designed application (app) and found that while their technology increased social interaction for 83% of the participants, only 33% felt more socially connected. Office et al.^30^ interviewed student volunteers who conducted one‐off phone calls during COVID‐19. Although negative effects were not reported, a positive effect on social isolation could not be identified from the evidence provided. Finally, Mountain et al.^33^ conducted an RCT using volunteers trained to telephone older people living independently, for 20 minutes per week over 10 weeks. They found small improvements in mental well‐being but no reported difference in loneliness. The following discussion of findings considers the results linked to the types of interventions used.

### 3.4 Internet‐based applications

Internet‐based applications were used in five studies.^25^, ^27^, ^28^, ^29^, ^39^ Guo et al.^29^ recorded participants using Internet‐based features such as social networking sites, Skype and audio calls. In contrast, Baez et al.^27^ trialled a purpose‐built simulated gym program that could be accessed through the Internet or phone/tablet application. Participants reported decreasing perceptions of loneliness and feelings of togetherness through messaging and virtual group activities where participants could connect with others. However, the most significant reductions in loneliness were among those with the highest initial perceived loneliness.^27^ Mellor, Firth and Moore equipped RACFs with communal computers with access to the Internet, email and chat rooms.^39^ Participants found adopting the new technology frustrating at times; however, the study found an overall improvement in social connectedness through Internet access, which peaked at 3 months and reduced at the 6‐ and 12‐month marks. Both Guo et al. and Czaja et al. found email was the most used feature and regarded as most valuable, whereas photo sharing and videoconferencing features were not as highly regarded.^25^, ^29^

Czaja et al.^25^ used a purpose‐built application, which engaged older people throughout the design process. Participants found the technology easy to use and gained confidence quickly. At 6 months, the intervention group reported significant decreases in loneliness and social isolation, and increased social support in comparison with the non‐technological intervention group. Although these changes were maintained at 12 months, the differences between the two groups did not remain the same. Those in the non‐technological intervention also reported decreases in loneliness and social isolation as they were provided with paper formats of similar activities offered on the technological interface and the opportunity to connect with other participants via mail.

Fokkema and Knipscheer^28^ loaned computers to participants for 3 years and found a significant reduction in loneliness, particularly among those who were more educated or reported the highest initial rates of loneliness.

### 3.5 Telephone support networks

Four studies specifically looked at the use of telephones, with mixed results.^30^, ^32^, ^33^, ^37^ Two studies, conducted during the COVID‐19 pandemic, engaged medical students to contact older people.^30^, ^37^ Office et al.^30^ conducted one‐off phone calls but was unable to draw conclusions as to their impact on social isolation or loneliness. Recurring phone calls were made by student volunteers in van Dyck et al.’s study.^37^ They found older people who were regularly contacted looked forward to their calls and the volunteers also felt more socially connected. Mountain et al. conducted an RCT using volunteers trained to telephone older people living independently, for 20 min per week over 10 weeks.^33^ They found small improvements in mental well‐being but no reported difference in loneliness; however, the high dropout rates of the volunteers meant that many older people were not contacted regularly. Finally, Cattan, Kime and Bagnall interviewed participants of a long‐term telephone befriending scheme, and reported high levels of satisfaction with the program and a reduced sense of social isolation.^32^

### 3.6 Applications for phones and tablets

Three studies used applications accessed through smartphones or tablets.^27^, ^31^, ^38^ Zaine et al.^31^ used media parcels to facilitate and guide photo and video sharing between friends and family members. They found participants reported increased frequency of interaction and improved depth of discussions, along with strengthened social connectedness. Barbosa Neves et al.^38^ developed an app with the input of older people, to provide messaging, and audio, photo and video sharing. Although participants initially reported difficulty using the app, the adoption of more complex features increased throughout the trial. Both apps significantly increased social interaction, but Barbosa Neves et al.^38^ found 66% of their participants did not report increased social connectedness.^31^ They also found those who felt more socially connected as a result of the intervention were more likely to have friends and family members living far away or overseas. The use of the app also declined from daily to weekly over time.

### 3.7 Videoconferencing

Videoconferencing was investigated in four studies.^26^, ^34^, ^35^, ^36^ The researchers used videoconferencing capabilities to link nursing home residents with family and friends.^34^, ^35^, ^36^ All of these studies reported positive, long‐term effects on loneliness in comparison with control groups and found that while the frequency of videoconference calls decreased over time, the length of calls increased. Tsai and Tsai was the only long‐term study (12 months) involving videoconferencing that identified an improvement in perceived loneliness.^36^ Tsai et al.^34^ suggest that their results could be attributed to the intervention requiring family and friends to contact participants and not the videoconferencing per se. Another qualitative trial, conducted in 2012, used purpose‐designed, remote‐controlled robots with videoconferencing capabilities, accessible by the participant and nominated connections (family members).^26^ The trial was only over 2 days, but 85% of participants responded positively, detailing feelings of safety and companionship, and family members found the ICT reassuring and calming.

### 3.8 Purpose‐designed applications

Of our included studies, five implemented purpose‐designed ICT,^25^, ^26^, ^27^, ^31^, ^38^ four of which positively affected social isolation and social connectedness.^21^, ^25^, ^26^, ^31^ Czaja et al.’s^25^ co‐designed application was described by participants as easy to adopt and use. Zaine et al.^31^ only reported minor technological difficulties relating to design aspects out of their control. Similarly, Seelye et al.’s^26^ challenges came from the usability of the remote‐controlled robot, not their ICT. Baez et al.^27^ alluded to discussions between a technician and participants relating to technical difficulties but did not disclose their content. Finally, Barbosa Neves et al.^38^ found that while their co‐designed ICT application increased social interactions, only 33% felt more socially connected and they reported that participants found it difficult to use initially.

### 3.9 Training and technical support

Studies using face‐to‐face training as their intervention were not included in this literature review; however, several studies provided some degree of assistance to older people adopting new technology. The level of assistance ranged from a 1.5‐hour training session predeployment of the ICT intervention^27^ or an initial training session with ongoing assistance.^39^ When assessing whether participants struggled with adopting the technology or not (excluding telephone interventions), those who had received training or technical support found adoption easier (7 of 11).^25^, ^27^, ^29^, ^31^, ^34^, ^35^, ^36^ Additionally, two studies found participants struggled to adopt the ICT when assistance was available,^38^, ^39^ one study reported similar experiences without offering assistance^26^ and another study did not provide training and reported no issues with ICT adoption.^28^

## 4 DISCUSSION

Overall, the studies included in this review found that most ICT interventions had some impact on the social isolation experienced by older people; however, as previous reviews have found,^3^, ^26^, ^27^ the heterogeneity of interventions (e.g., from videoconferencing robots to telephones), and variety of outcome measures, limits strong conclusions being drawn about the effectiveness of different interventions. In line with Barbosa Neves et al.,^38^ this review was not able to identify any specific features of the ICT interventions (e.g., instant messaging versus email) that had a greater impact on isolation than others, although audio or picture/video messages were valued by older participants for providing visual cues.^40^ To mimic the impact of mandated periods of isolation, such as during the current pandemic, this review attempted to focus on research investigating ICT interventions that did not require face‐to‐face training or interactions. Again, the conclusions that can be drawn about the effectiveness of ICT interventions within this context are limited; however, drawing from the findings, the following discussion highlights a series of recommendations that can inform the implementation of ICT during periods of mandated isolation.

### 4.1 Recommendations

#### 4.1.1 Provide training and technical support

The review found that many older people resisted adoption of ICT due to difficulty or limited understanding of how to engage with the technology. Currently, 75% of digitally disengaged Australians are older than 70,^41^ with research conducted during the COVID‐19 pandemic identifying limited technological support as one of the most critical barriers impacting access for older people.^42^, ^43^ In line with this, we recommend that training and ongoing technical support be available for older people adopting new ICTs. However, novel approaches that address the barriers of mandated isolation must be identified, for example engaging staff assistance where possible, or involving younger–older people (60–75) to assist older people (75+).^44^ Where family visits are possible, families can also be engaged to support training and uptake.^45^, ^46^

#### 4.1.2 Prioritise co‐designed and purpose‐designed ICT

A second recommendation calls for ICT to be co‐designed or co‐implemented with older people. Our review identified some studies that used purpose‐built and/or co‐designed technology, which was well received by participants.^25^, ^26^, ^29^, ^31^ Haase et al.^45^ have also found older people were more willing to adopt new technology during the COVID‐19 pandemic when it was perceived to be accessible and user‐friendly. The engagement of older people as key stakeholders in all stages of technology selection and application should be a minimum requirement for any ICT intervention. This engagement prioritises interface acceptability and usability for older people and, in the case of purpose‐built technology, leads to intuitive and simple designs that can potentially minimise difficulty and confusion linked to the adoption of new technologies.^26^, ^38^, ^39^, ^47^, ^48^, ^49^ Importantly, the processes of engagement must be central and ongoing. The high uptake of ICT during the COVID‐19 pandemic has created an unprecedented opportunity for researchers to work in collaboration with older people to evaluate current technologies and interventions. Videoconferencing applications (e.g., Zoom, Facetime and Microsoft Teams) provide an important case in point as they have been a key resource used by many during the pandemic.^43^ However, current evidence examining the acceptability of videoconferencing applications for older people is limited, and understanding of their impact on social isolation is not well understood^3^, ^19^, ^40^ This gap in research needs to be filled urgently, but in doing so, researchers must use this opportunity to build stakeholder engagement and inform intervention design and implementation outside the pressures of mandated isolation.

#### 4.1.3 Invite family members to engage

Another consistent observation from our review was the social and emotional benefits experienced by family members, spouses and friends who were able to maintain contact with their older relatives through ICT interventions.^26^, ^29^, ^31^ Family members have previously reported the negative effects of miscommunication, or lack thereof, between themselves, residents and RACFs.^50^, ^51^ During COVID‐19 lockdowns, the effects of miscommunication were amplified when families were unable to contact their loved ones, causing anger, stress and anxiety.^52^ In contrast, our review found family members experienced feelings of reassurance, calmness and reduced anxiety when technological interventions were available to facilitate contact.^26^, ^29^, ^31^ Equipping older people with the skills and ICT to connect with family members has the potential to alleviate distress during times of enforced isolation, while also increasing opportunities for connection at other times. In line with this, and consistent with our recommendation for the engagement of older people as key stakeholders, we also recommend that families and friends be engaged in the implementation and roll‐out of ICT interventions, wherever possible, to support uptake and facilitate connection. In presenting this recommendation, it is acknowledged that social isolation may stem from the absence of family and/or friends, but in such instances, interventions involving contact with others, including trained volunteers,^33^, ^37^ could be explored. Such an approach would need to manage carefully the high volunteer dropout rates identified in some interventions in this review,^33^, ^37^ with investment needed to maximise the sustainability of the volunteer workforce and ensure supportive relationships are fostered.

#### 4.1.4 Encourage ICT use for social connection during and outside periods of increased isolation

Although the body of evidence is small, some studies reported those experiencing the highest rates of social isolation gained the most from ICT interventions in terms of social connection.^27^, ^28^ This suggests that increased experiences of isolation may be linked to great ICT uptake as people seek out opportunities for increased social interaction.^27^, ^28^, ^38^ Therefore, we recommend ongoing use of and support for a range of ICT and systems to enable rapid implementation of digital social connectivity to occur if and when required. This additional way of connecting not only supports people during mandated isolation but also enables increased social connection and fostering of relationships at other times, including during periods of hospitalisation or illness recovery. However, in proposing this recommendation, we also provide a note of caution. The findings suggest ICT should not be relied on to prevent social isolation in the long term.^36^, ^39^, ^50^ In line with this, we recommend regular assessments be conducted to ensure older people, family members or RACFs do not become dependent or reliant on ICT as a principal method of social connection or as an alternative to face‐to‐face interactions.^36^

#### 4.1.5 Limitations

Several limitations impact this review. In particular, the literature search was limited to health‐related databases and the scanning of key reference lists. In focusing on health‐related literature, the search strategy may not have incorporated publications from other relevant fields, including the human–computer interaction field. To improve the strength of further research, a broader database search along with detailed consideration of grey literature and evaluation studies drawn from the experiences of different services and facilities supporting older people should be considered. Our search strategy also only included studies published from 2000 in OECD countries, although some of the technology analysed was available before this date, so it is possible that some older technologies that are still current may be suitable for the settings we have considered. Finally, in seeking to provide a breadth of understanding across the settings in which older people live, we included RACFs, private homes and palliative care in our search. Our ability to provide strong conclusions on the effectiveness of interventions may have been hampered by the inclusion of multiple settings, each of which has varying levels of support available. Future research should look to restrict consideration to settings that have comparable resources.

## 5 CONCLUSIONS

Overall, we found evidence of ICT improving social connectedness of older people to some extent although more rigorous research in future is needed. Recommendations from previous literature highlight the importance of including older people in purposeful design, engaging families and support networks, and providing ongoing ICT training and support so that systems and skills are in place for future periods of mandated isolation. The literature also warns us not to rely on ICT as the only avenue for social interaction either during or outside periods of social distancing.

## CONFLICTS OF INTEREST

No conflicts of interest declared.

## Data Availability

No new data were created for this study, therefore data sharing is not applicable to this article.
